# *OsSCL30* overexpression reduces the tolerance of rice seedlings to low temperature, drought and salt

**DOI:** 10.1038/s41598-022-12438-4

**Published:** 2022-05-19

**Authors:** Jia Zhang, Yihao Sun, Zhanmei Zhou, Yifan Zhang, Yanmei Yang, Xiaofei Zan, Xiaohong Li, Jiale Wan, Xiaoling Gao, Rongjun Chen, Zhengjian Huang, Lihua Li, Zhengjun Xu

**Affiliations:** Crop Ecophysiology and Cultivation Key Laboratory of Sichuan Province, Rice Research Institute of Sichuan Agricultural University, Chengdu, 611130 Sichuan China

**Keywords:** Physiology, Plant sciences

## Abstract

Rice is one of the main food crops for the world population. Various abiotic stresses, such as low temperature, drought, and high salinity, affect rice during the entire growth period, determining its yield and quality, and even leading to plant death. In this study, by constructing overexpression vectors D-163 + 1300:*OsSCL30* and D-163 + 1300-AcGFP:*OsSCL30-GFP*, the mechanism of action of *OsSCL30* in various abiotic stresses was explored. Bioinformatics analysis showed that *OsSCL30* was located on the chromosome 12 of rice Nipponbare, belonging to the plant-specific SCL subfamily of the SR protein family. The 1500 bp section upstream of the open reading frame start site contains stress-related cis-acting elements such as ABRE, MYC, and MYB. Under normal conditions, the expression of *OsSCL30* was higher in leaves and leaf sheaths. The results of reverse transcription polymerase chain reaction showed that the expression of *OsSCL30* decreased after low temperature, drought and salt treatment. In root cells *OsSCL30* was localized in the nuclei. The results of the rice seedling tolerance and recovery tests showed that overexpression of *OsSCL30* diminished the resistance to low temperature, drought and salt stresses in transgenic rice and resulted in larger accumulation of reactive oxygen species. This study is of great significance for exploring the response mechanisms of SR proteins under abiotic stresses.

## Introduction

Rice (*Oryza sativa L.*) is one of the main food crops, and about 50% of the world’s population depends on rice as the staple food^1,2^. Plants may encounter various abiotic stresses during their growth and development, among which low temperature, drought and salinity are common stress conditions that determine their yield and quality, and may even lead to plant death^3–6^. Rice, when subjected to abiotic stresses, responds and adapts at the molecular, cellular, biochemical and physiological levels to survive under stress conditions^7–9^.

A family of serine/arginine (SR) -rich proteins plays important roles in both constitutive and alternative splicing by binding to specific RNA sequences and assembling spliceosomes at weakly spliced sites for alternative splicing^10–12^. The sequence feature of the canonical SR protein family is the presence of one or two N-terminal RNA recognition motifs (RRMs), followed by a serine/arginine-rich repeat domain of at least 50 amino acids. The arginine/serine (RS) or SR repeats are characterized by an RS content greater than 40%. Stresses such as low temperature, high temperature, and drought regulate the splicing pattern, phosphorylation state, and subcellular distribution of SR proteins in plants^13^. The SR proteins of wheat (*Triticum aestivum*) and brachypodium (*Brachypodium distachyon*) are similar to the rice SR proteins and can be widely expressed. In the promoter regions of SR protein genes of wheat and brachypodium, 92 cis-elements related to plant growth and development, stress and hormone were found, indicating that SR proteins play an important role in plant growth and development and stress response^14–17^.

Low temperature stress is a major limiting factor for rice growth and geographic distribution, affecting both vegetative and reproductive stages, influencing seed germination, seedling growth, plant height, photosynthesis, heading date, and fertility^18–20^. Many transcription factors (TFs) play a role in low temperature stress, such as MYB4, MYBS3, *OsNAC5*, and *OsbZIP73*^21–23^. Three rice CBF gene family members (*OsCBF1*, *OsCBF2,* and *OsCBF3*) were up-regulated under low temperature stress^24^, these three CBF proteins are the major transcriptional activators required for cold-induced gene expression^25^. Rice has at least four DREB2 homologous genes, of which *OsDREB2A* and *OsDREB2B* are induced by drought, high salt and high temperature stresses. The expression of *OsDREB2B* is regulated by alternative splicing, resulting in two types of transcripts: functional and non-functional forms^26^.

The role of SR proteins in plant growth, development and stress response has been studied. For example, the SCL30a from cassava (*Manihot esculenta*) overexpressed in *Arabidopsis* negatively regulates salt tolerance^27^; and deletion of SR45 in *Arabidopsis* results in enhancing sensitivity to salt stress and altering the expression and splicing of genes which regulate salt stress response^28^. Currently, rice growth is affected by environmental stresses such as salinity, drought, and extreme temperature. Therefore, it is of great significance to understand the response of SR proteins in rice under abiotic stress. In the present study, the results showed that overexpression of *OsSCL30* diminished the resistance to low temperature, drought and salt in transgenic rice.

## Results

### Bioinformatics analysis of *OsSCL30*

**Figure 1:**
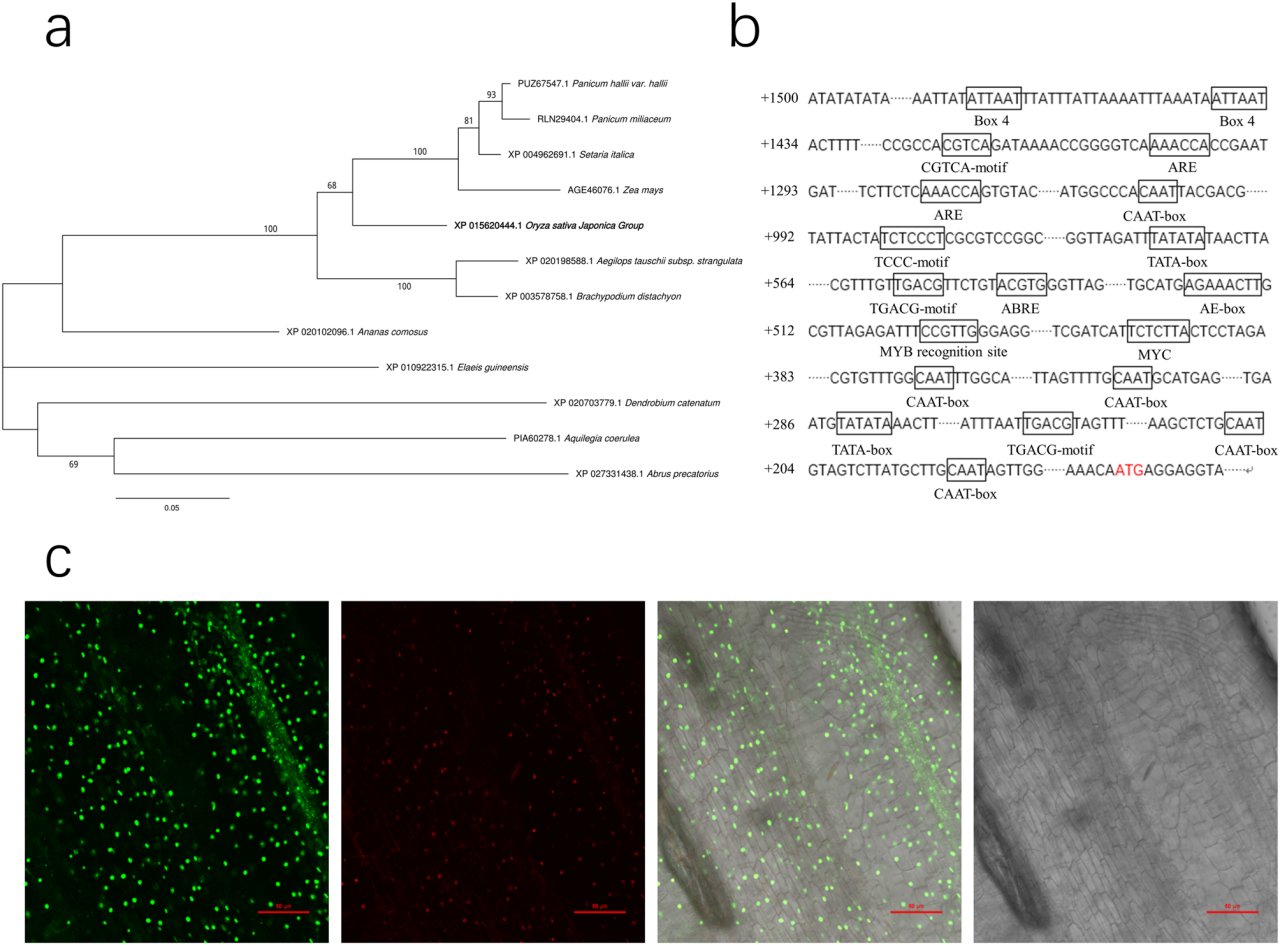
(**a**) Cluster analysis of *OsSCL30* homologous genes in plants. (**b**) Analysis of elements associated with the *OsSCL30* promoter region. (**c**) Subcellular localization of *OsSCL30-GFP*.

The *OsSCL30* gene is located on rice chromosome 12, with an open reading frame of 792 bp, encoding 263 amino acids. It belongs to the plant-specific SCL subfamily of the SR protein family. In order to understand the relationship of *OsSCL30* sequences among different species, the species with high amino acid sequence similarity were selected for cluster analysis. The *OsSCL30* was found to be clustered with panicgrass (*Panicum hallii*), broomcorn millet (*Panicum miliaceum*), millet (*Setaria italica*), and maize (*Zea mays*), with a strong genetic relationship (Fig. 1a). Using the PlantCARE online website to analyze about 1500 bp upstream of ATG, it was found that the promoter of the *OsSCL30* gene contains various elements related to stress response (Fig. 1b). These elements include ABRE (ABA responsive element), G-box (light-responsive element), TGA-element (auxin-responsive element), GCN4_motif (involved in endosperm expression), TC-rich repeats (involved in defense and stress response), MYB (drought-related response element), etc.

### Relative expression of *OsSCL30*

**Figure 2:**
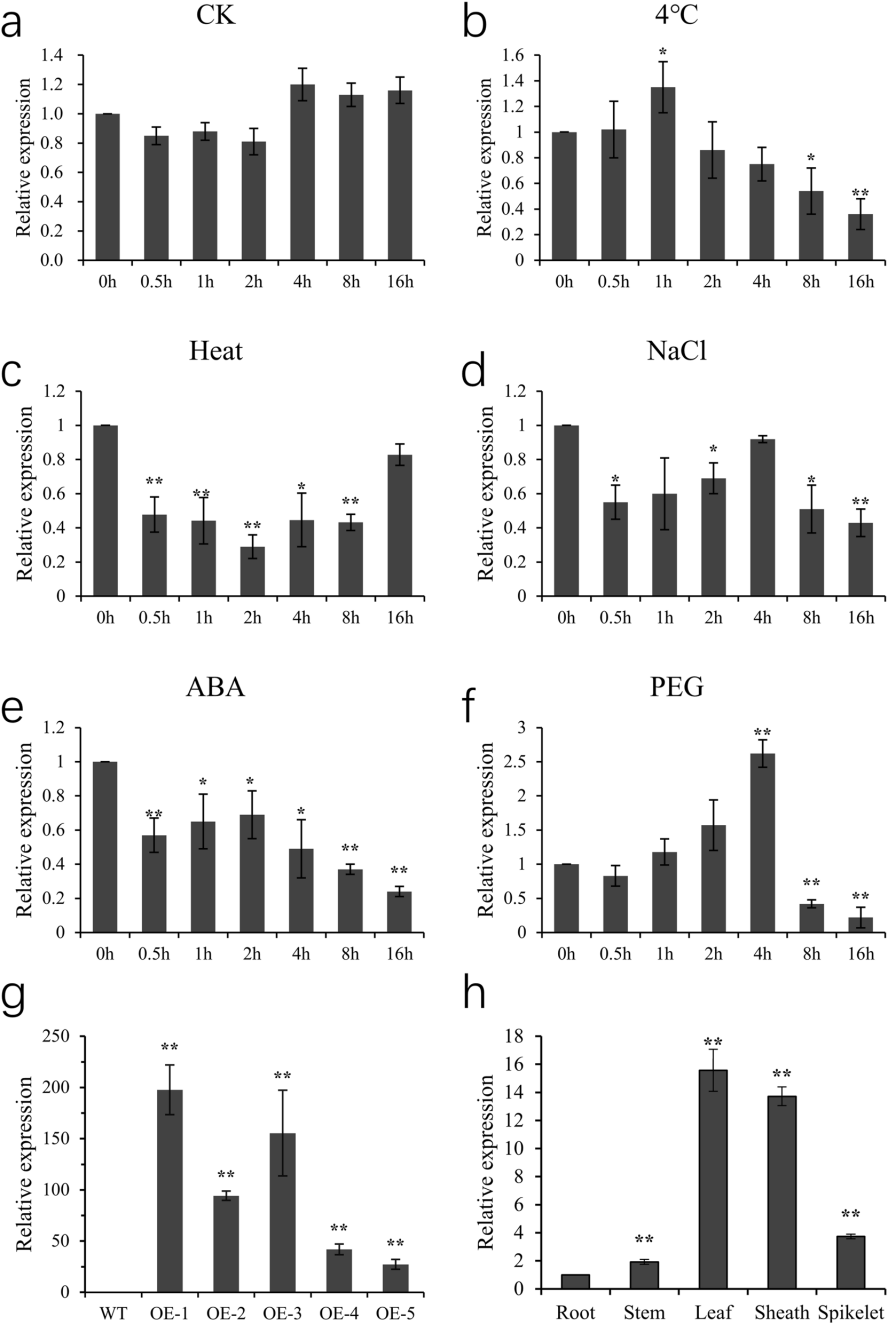
(**a**–**f**) *OsSCL30* gene expression pattern under various environmental stresses and ABA treatment. (a. ck; b. 4 °C; c. 42 °C; d. 150 mM NaCl; e. 50 μM ABA; f. 15% w/v PEG) (**g**) *OsSCL30* transgenic lines expression detection. (**h**) Tissue expression of *OsSCL30*. Error bars represent ± SE (n = 3). Asterisks indicate significant differences (*P < 0.05, **P < 0.01).

In order to select overexpressed lines for subsequent experiments, the expression level of *OsSCL30* was detected by qRT-PCR. The results (Fig. 2g) showed that compared with WT, the expression levels of OE-1 and OE-3 were up-regulated the most, which were 197 times and 155 times, respectively. Therefore, OE-1 and OE-3 were selected as the subsequent experimental lines.

To detect the tissue-specific expression pattern of *OsSCL30* gene, different untreated tissues (root, stem, leaf, sheath and spikelet) were collected, and the expression level of *OsSCL30* in different tissues was detected by qRT-PCR. The results (Fig. 2h) showed the expression of *OsSCL30* was highest in leaves, followed by leaf sheaths.

### *OsSCL30* is activated by abiotic stress

Because a variety of stress response factors were found in the promoter of *OsSCL30*, different stresses were applied to rice seedlings, and the expression level of *OsSCL30* was detected by qRT-PCR. The results showed that the expression level of *OsSCL30* in the control group was similar to the initial value during the whole process (Fig. 2a). The expression of *OsSCL30* increased briefly after the low temperature treatment was imposed, rising to the highest level at 1 h, and then gradually decreased (Fig. 2b). The change in the whole period was not significant. The expression of *OsSCL30* began to decrease immediately after imposition of the high temperature treatment, decreasing to a minimum at 2 h, and then gradually increased, but was lower than the initial value throughout the treatment (Fig. 2c). Under salt stress, the expression of *OsSCL30* decreased significantly, then increased with the treatment duration, reaching the maximum at 4 h and then began to decrease, but the expression was lower than the initial value during the whole treatment duration (Fig. 2d). Under exogenous ABA treatment, the expression level of *OsSCL30* was similar to that under salt treatment (Fig. 2e). After 0.5 h of drought treatment, the expression of *OsSCL30* decreased briefly, then increased gradually, reaching a peak at 4 h, and then decreased sharply with prolonged treatment duration (Fig. 2f). In summary, various abiotic stresses can reduce the expression of *OsSCL30* gene; among them, drought stress has the greatest impact on the *OsSCL30* expression.

### Subcellular localization of *OsSCL30*

The WOLF PSORT online website predicts that the *OsSCL30* protein is localized in the nucleus. To further examine the subcellular localization of *OsSCL30* protein in rice, the roots of transgenic rice plants *OsSCL30-GFP* were stained with DAPI and observed by confocal microscopy. The results showed that the GFP signal of the SCL30-GFP fusion protein was visible only in the nucleus, consistent with the prediction (Fig. 1c). Hence, we concluded *OsSCL30* protein was localized in the nucleus of rice.

### Overexpression of *OsSCL30* attenuates low temperature resistance in transgenic rice

**Figure 3:**
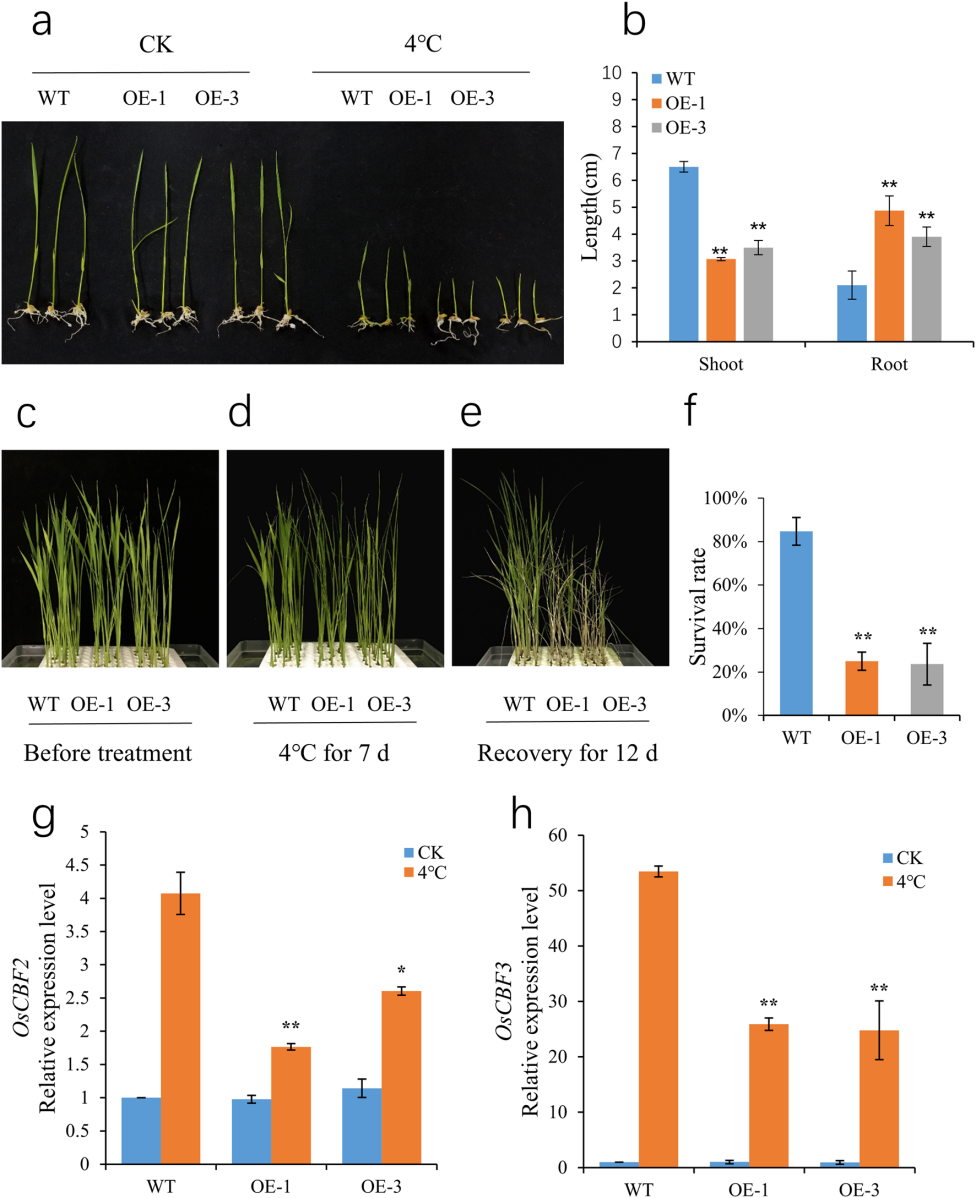
Effects of cold stress in wild type (WT) and OE lines. (**a**) Phenotypes of WT and OE lines under 4 °C for 3 days. (**b**) Plant height and root length of WT and OE lines treated at 4 °C for 3 days. (**c**) Phenotype of WT and OE lines hydroponically cultivated for 14 days (before imposition of the cold stress). (**d**) Phenotypes of WT and OE lines treated for 7 days at 4 °C. (**e**) Phenotypes of WT and OE lines after 12 days recovery following the 4 °C treatment. (**f**) Survival of recovered WT and OE lines. (**g**–**h**) The expression levels of *OsCBF2* and *OsCBF3* were analyzed after two days of treatment at 4 °C, respectively. Error bars represent ± SE (n = 3). Asterisks indicate significant differences between transgenic lines and WT (*P < 0.05, **P < 0.01).

To verify the response of *OsSCL30* overexpression to low temperature stress, we cultured 5-day-old Nip (WT) and two overexpression lines (OE-1, OE-3) at 4 °C for 3 days followed by 7-day recovery. The results showed that the growth of *OsSCL30-OE* was similar to that of WT seedlings under normal growth conditions, but low temperature inhibited the growth of *OsSCL30-OE* seedlings (Fig. 3a). The plant height of *OsSCL30-OE* seedlings after low temperature treatment was significantly shorter than that of WT, but the root length was significantly longer than that of WT (Fig. 3b).

We cultured 14-day-old Nip (WT) and two overexpressing lines (OE-1, OE-3) at 4 °C for 7 days and then allowed them to recover for 12 days. The wilting of *OsSCL30-OE* seedlings after low temperature treatment was more severe than that of WT (Fig. 3d). After the recovery period, the survival rate of *OsSCL30-OE* seedlings (24–25% for the two lines) was significantly lower than that of WT (85%) (Fig. 3e–f).

In order to clarify the regulatory pathway of *OsSCL30*, we detected the expression of important genes in the cold response pathway of *OsSCL30* overexpressed plants. The results showed that the core components of rice ICE-CBF pathway^29^, including *OsCBF2* and *OsCBF3*, were down regulated in *OsSCL30-OE* compared with WT (Fig. 3g–h). In conclusion, overexpression of *OsSCL30* reduced the low temperature resistance of transgenic rice plants.

### Overexpression of *OsSCL30* attenuates drought resistance in transgenic rice

**Figure 4:**
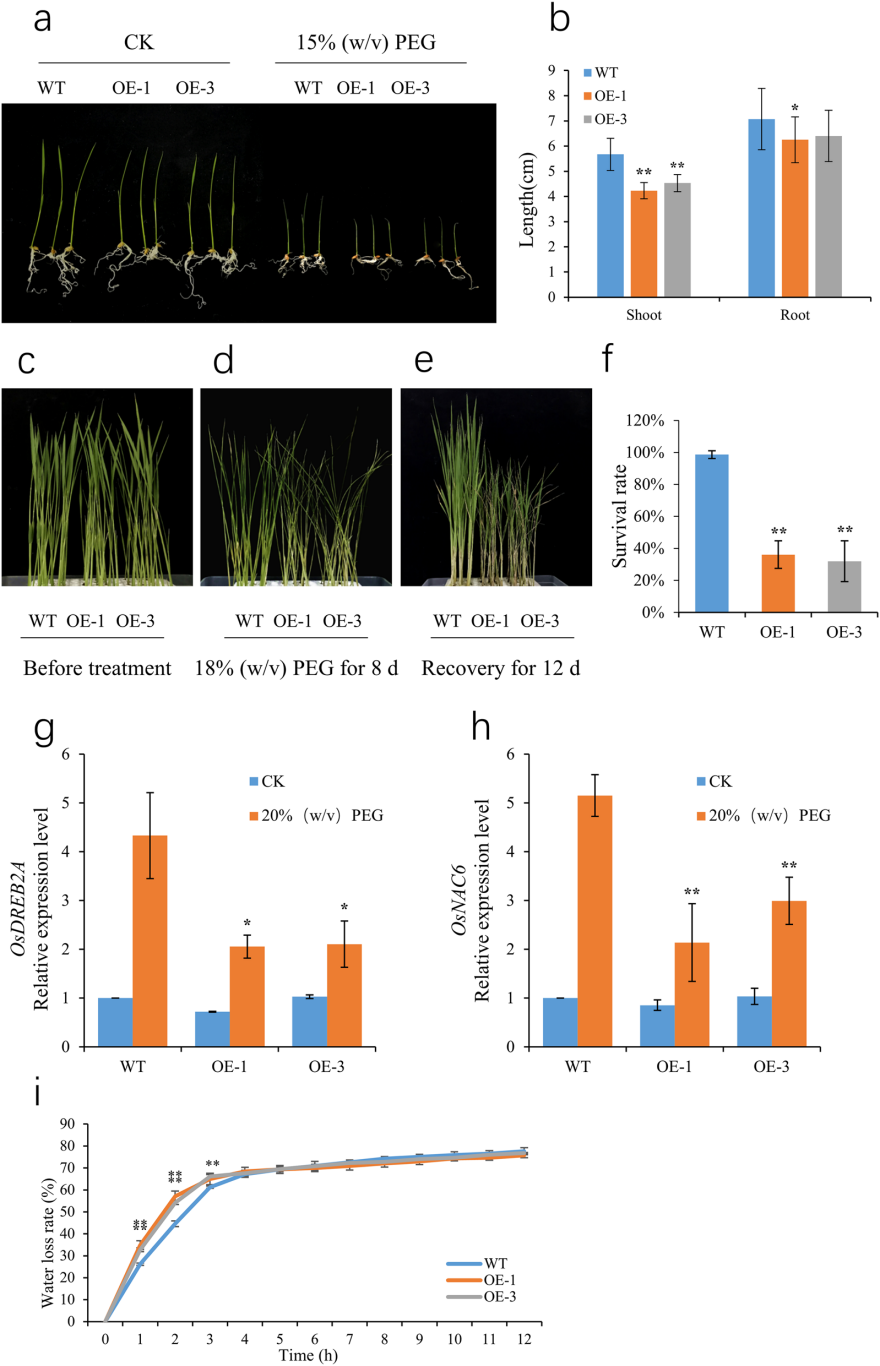
Effects of drought stress in wild type (WT) and OE lines. (**a**) Phenotypes of WT and OE lines under 15% w/v PEG for 3 days. (**b**) Plant height and root length of WT and OE lines treated at 15% w/v PEG for 3 days. (**c**) Phenotype of WT and OE lines hydroponically cultivated for 14 days (before imposition of the drought stress). (**d**) Phenotypes of WT and OE lines treated for 8 days at 18% w/v PEG. (**e**) Phenotypes of WT and OE lines after 12-day recovery following the 18% w/v PEG treatment. (**f**) Survival of recovered WT and OE lines. (**g**–**h**) The expression levels of *OsDREB2A* and *OsNAC6* were analyzed after two days of treatment at 20% w/v PEG, respectively. (**i**) Water loss rates of detached leaves from 14-day-old plants. Error bars represent ± SE (n = 3). Asterisks indicate significant differences between transgenic lines and WT (*P < 0.05, **P < 0.01).

To verify the response of *OsSCL30* overexpression to drought stress, we cultured 5-day-old Nip (WT) and two overexpressing lines (OE-1, OE-3) in 15% w/v PEG for 3 days followed by 7-day recovery. The *OsSCL30-OE* grew similarly to WT seedlings under normal growth conditions, but drought inhibited the growth of *OsSCL30-OE* seedlings (Fig. 4a). After drought treatment, the plant height and root length of transgenic lines were shorter (root length was significant in OE-1 and not significant in OE-3) compared with WT (Fig. 4b).

To exacerbate the drought stress, we cultured 14-day-old Nip (WT) and two overexpressing lines (OE-1, OE-3) in 18% w/v PEG environment for 8 days and then allowed them to recover for 12 days. The results showed that the severity of wilting was similar in *OsSCL30-OE* and WT seedlings after 8 days of 18% w/v PEG treatment (Fig. 4d). However, after the recovery period, the survival rate of *OsSCL30-OE* seedlings (32–36% for the two OE lines) was significantly lower than that of WT (98%) (Fig. 4e–f).

In order to explore the regulatory mechanism of *OsSCL30* under drought stress, we detected the expression of important genes in the drought response pathway of *OsSCL30* overexpressing plants. The results showed that the expression of drought related response genes *OsDREB2A*^30^ and *OsNAC6*^31^ in rice *OsSCL30-OE* was down regulated compared with WT (Fig. 4g–h).

In addition, compared with WT, the water loss rate of detached leaves of *OsSCL30-OE* was significantly higher, indicating that the water holding capacity of *OsSCL30-OE* was weaker (Fig. 4i). In conclusion, overexpression of *OsSCL30* reduced the drought resistance of transgenic rice plants.

### Overexpression of *OsSCL30* weakens salt resistance in transgenic rice

**Figure 5:**
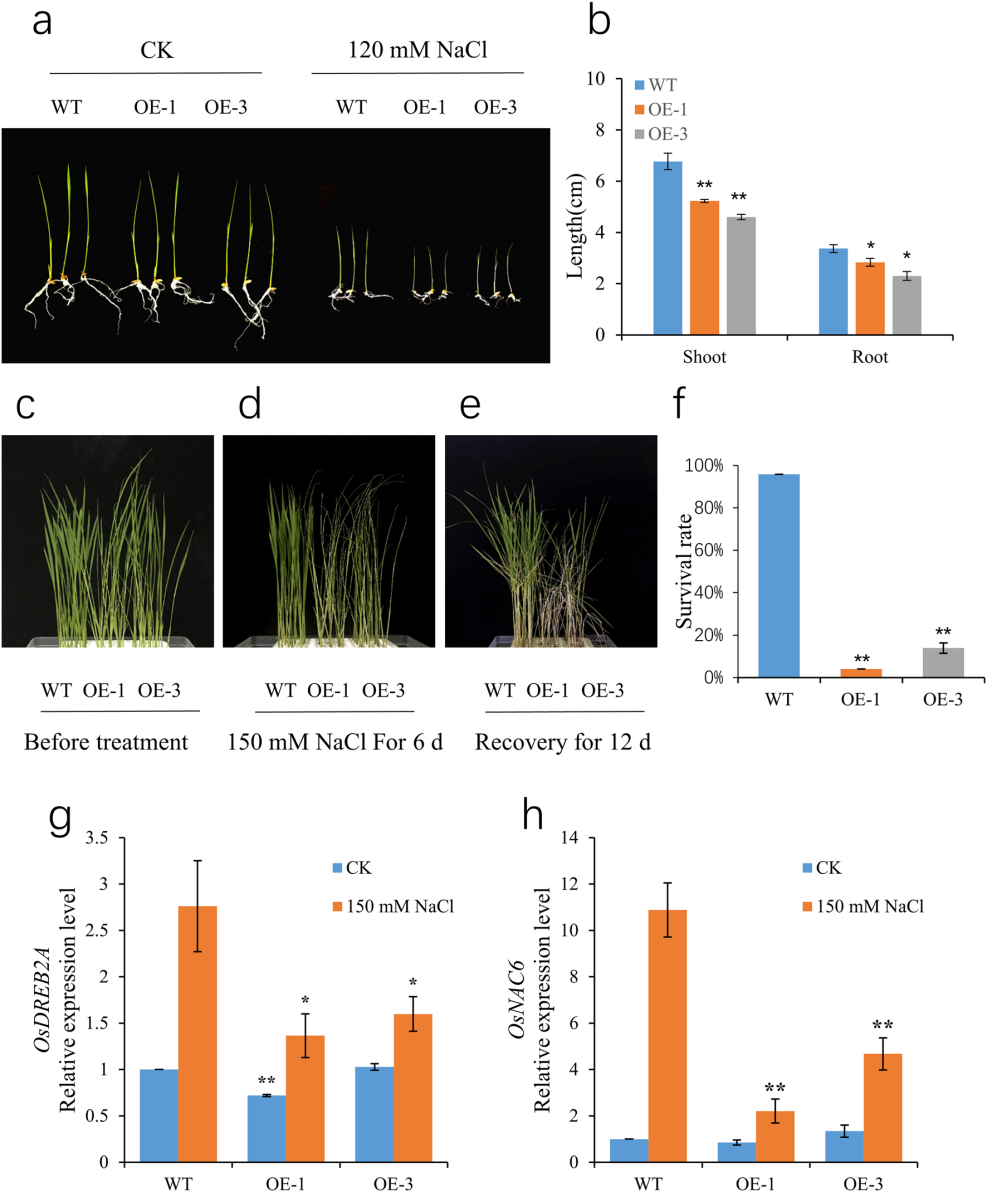
Effects of salt stress in wild type (WT) and OE lines. (**a**) Phenotypes of WT and OE lines under 120 mM NaCl for 3 days. (**b**) Plant height and root length of WT and OE lines treated at 120 mM NaCl for 3 days. (**c**) Phenotype of WT and OE lines hydroponically cultivated for 14 days (before imposition of the salt stress). (**d**) Phenotypes of WT and OE lines treated for 6 days at 150 mM NaCl. (**e**) Phenotypes of WT and OE lines after 12 days recovery following the 150 mM NaCl treatment. (**f**) Survival of recovered WT and OE lines. (**g**–**h**) The expression levels of *OsDREB2A* and *OsNAC6* were analyzed after two days of treatment at 150 mM NaCl, respectively. Error bars represent ± SE (n = 3). Asterisks indicate significant differences between transgenic lines and WT (*P < 0.05, **P < 0.01).

To verify the response of *OsSCL30* overexpression to high salt stress, we cultured 5-day-old Nip (WT) and two overexpressing lines (OE-1, OE-3) in 120 mM NaCl for 3 days followed by recovery for 7 days. The *OsSCL30-OE* grew similarly to WT seedlings under normal growth conditions, but high salt inhibited the growth of *OsSCL30-OE* seedlings (Fig. 5a). After salt treatment, both plant height and root length of *OsSCL30-OE* seedlings were significantly shorter than those of WT (Fig. 5b).

We cultured 14-day-old Nip (WT) and two overexpressing lines (OE-1, OE-3) in 150 mM NaCl for 6 days and then allowed them to recover for 12 days. The results showed that wilting after 6 days of 150 mM NaCl treatment was more severe in *OsSCL30-OE* than WT seedlings (Fig. 5d). After recovery, the survival rate of *OsSCL30-OE* seedlings (4–14% for the two lines) was significantly lower than that of WT (96%) (Fig. 5e–f).

In order to explore the regulatory mechanism of *OsSCL30* under salt stress, we detected the expression of important genes in the salt response pathway of *OsSCL30* overexpressing plants. The results showed that the expression of salt related response genes *OsDREB2A*^30^ and *OsNAC6*^31^ in rice *OsSCL30-OE* was down regulated compared with WT (Fig. 5g–h). Therefore, we conclude the overexpression of *OsSCL30* reduced the salt tolerance of transgenic rice plants.

### Overexpression of *OsSCL30* affects the accumulation and scavenging of ROS under different stresses

**Figure 6:**
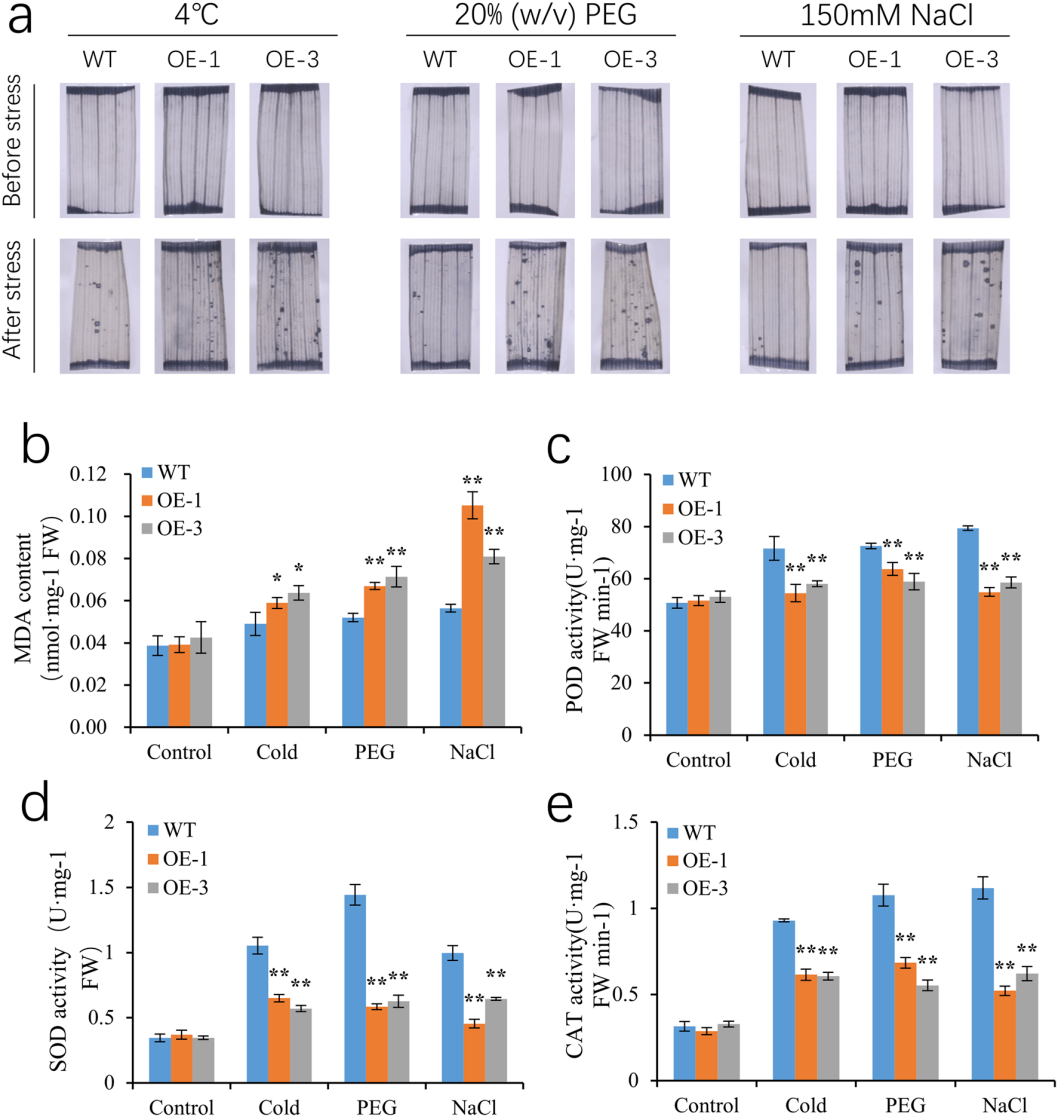
(**a**) Nitroblue tetrazolium (NBT) staining was used to detect the levels of superoxide anion in wild type (WT) and OE lines before and after low temperature (4 °C for 2 days), drought (20% w/v PEG for 2 days), and salt (150 mM NaCl for 2 days) treatments. (**b**–**e**) Detection of reactive oxygen species scavenging enzyme activities in wild type (WT) and OE lines before and after low temperature (4 °C for 2 days), drought (20% w/v PEG for 2 days) and salt (150 mM NaCl for 2 days). Error bars represent ± SE (n = 3). Asterisks indicate significant differences between transgenic lines and WT (*P < 0.05, **P < 0.01).

To examine the effect of *OsSCL30* overexpression on the accumulation of reactive oxygen species (ROS), we subjected 14-day-old Nip (WT) and two overexpressing lines (OE-1, OE-3) to different stresses, and performed NBT staining to estimate the accumulation of superoxide ions (O^2−^). In the control (no stress) treatment, the *OsSCL30-OE* leaves showed no significant difference from WT; by contrast, after the low temperature, drought and salt treatments the *OsSCL30-OE* leaves had numerous black spots (indicating more ROS accumulation and more severe oxidative damage) compared with WT (Fig. 6a). As an indication of cellular oxidative damage, the content of MDA showed no significant difference between *OsSCL30-OE* and WT in the control treatment without stress, whereas after low temperature, drought and salt treatments *OsSCL30-OE* accumulated more MDA and suffered more severe oxidative damage than WT (Fig. 6b).

The activities of reactive oxygen species scavenging enzymes (SOD, CAT and POD) were similar between *OsSCL30-OE* and WT without any stress imposed, whereas these activities were significantly lower in *OsSCL30-OE* than WT after the low temperature, drought and salt treatments, indicating relatively poor capacity of the *OsSCL30-OE* lines to scavenge reactive oxygen species (Fig. 6c–e).

**Figure 7:**
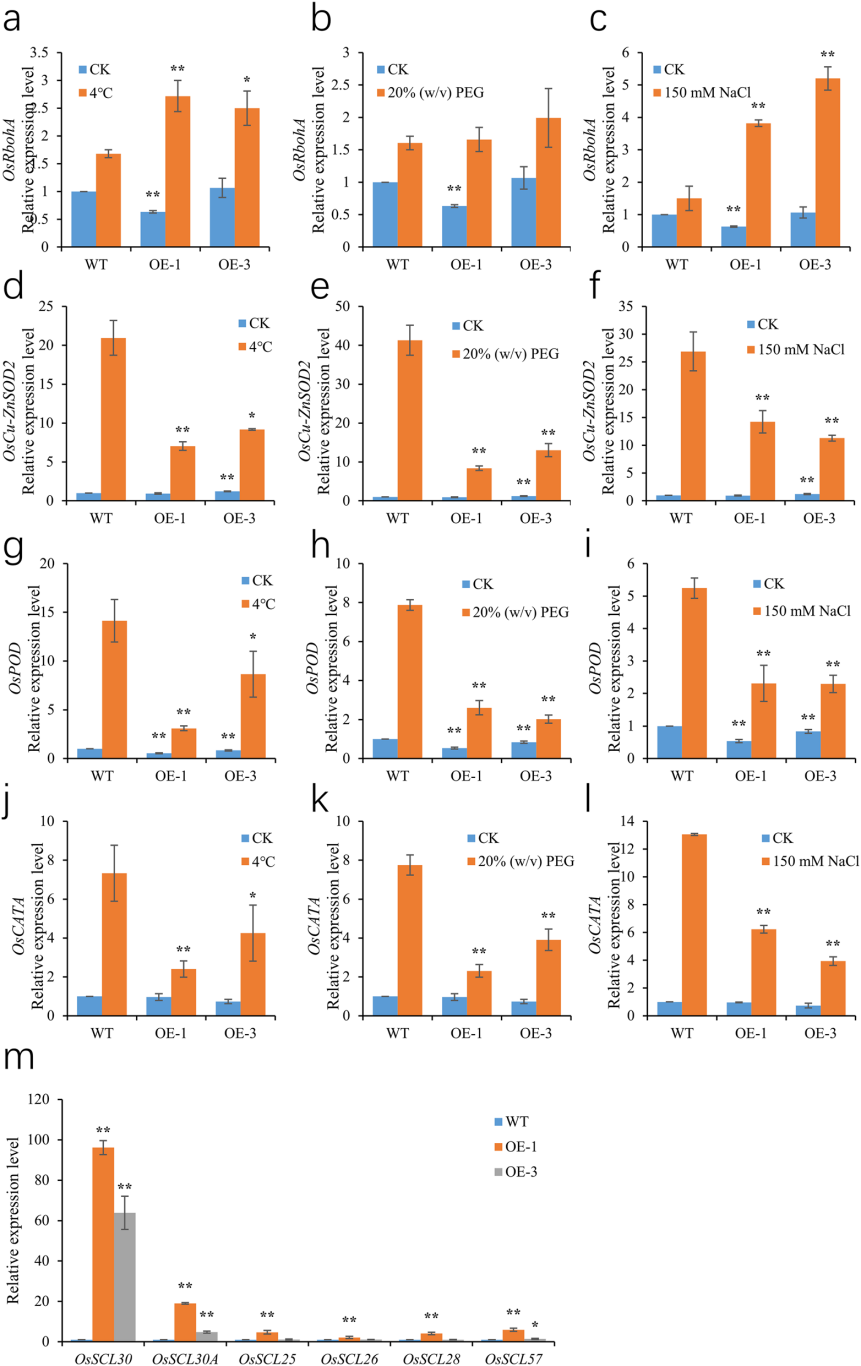
(**a**-**l**) Expression of ROS production and clearance related genes in WT and *OsSCL30-OE* plants. (**a**–**c**) Expression of *OsRbohA* in WT and *OsSCL30-OE* plants are treated with or without 4 °C, 20% w/v PEG and 150 mM NaCl for 2 days. (**d**–**f**) Expression of *OsCu-ZnSOD2* in WT and *OsSCL30-OE* plants are treated with or without 4 °C, 20% w/v PEG and 150 mM NaCl for 2 days. (**g**–**i**) Expression of *OsPOD* in WT and *OsSCL30-OE* plants are treated with or without 4 °C, 20% w/v PEG and 150 mM NaCl for 2 days. (**j**–**l**) Expression of *OsCATA* in WT and *OsSCL30-OE* plants are treated with or without 4 °C, 20% w/v PEG and 150 mM NaCl for 2 days. (m) Expression of SCL subfamily related genes in WT and *OsSCL30-OE* plants. Error bars represent ± SE (n = 3). Asterisks indicate significant differences between transgenic lines and WT (*P < 0.05, **P < 0.01).

In addition, we also analyzed the changes in expression levels of genes related to reactive oxygen species production and scavenging under cold, drought, salt stress and normal conditions, including *OsRbohA* (NADPH oxidase), *OsCu-ZnSOD2* (superoxide dismutase), *OsPOD* (peroxidase) and *OsCATA* (catalase)^32,33^. Under normal conditions, there was no significant difference in the expression of *OsRbohA*, *OsCu-ZnSOD2*, *OsPOD* and *OsCATA* between WT and *OsSCL30-OE*. However, under stress treatment, compared with WT, the expression of *OsRbohA* in *OsSCL30-OE* increased in varying degrees (Fig. 7a–c), and the expression of *OsCu-ZnSOD2*, *OsPOD* and *OsCATA* decreased in varying degrees (Fig. 7d–l). In conclusion, under low temperature, drought and salt stresses, overexpression of *OsSCL30* can reduce the activity of enzymes scavenging reactive oxygen species, resulting in aggravated oxidative damage.

### Relative expression of *OsSCL30* homologous proteins

It is an established fact that members of SR protein gene family play a crucial role in regulating proteome diversity in spatio-temporal manner. Since the SR protein family consists of multiple members, we studied the effect of *OsSCL30* overexpression on other SCL subfamilies in transgenic lines (*OsSCL30A*, *OsSCL25*, *OsSCL26*, *OsSCL28*, *OsSCL57*)^34^. The results showed that the expression of its homologous proteins increased, and the increase of *OsSCL30A* was the most significant (Fig. 7m). The overexpression of *OsSCL30* negatively regulated the abiotic stress tolerance of plants, which may be due to crosstalk with other members of the family, and the cumulative effect leaded to the sensitivity to abiotic stress.

### Analysis of agronomic traits in transgenic rice overexpressing *OsSCL30*

**Figure 8:**
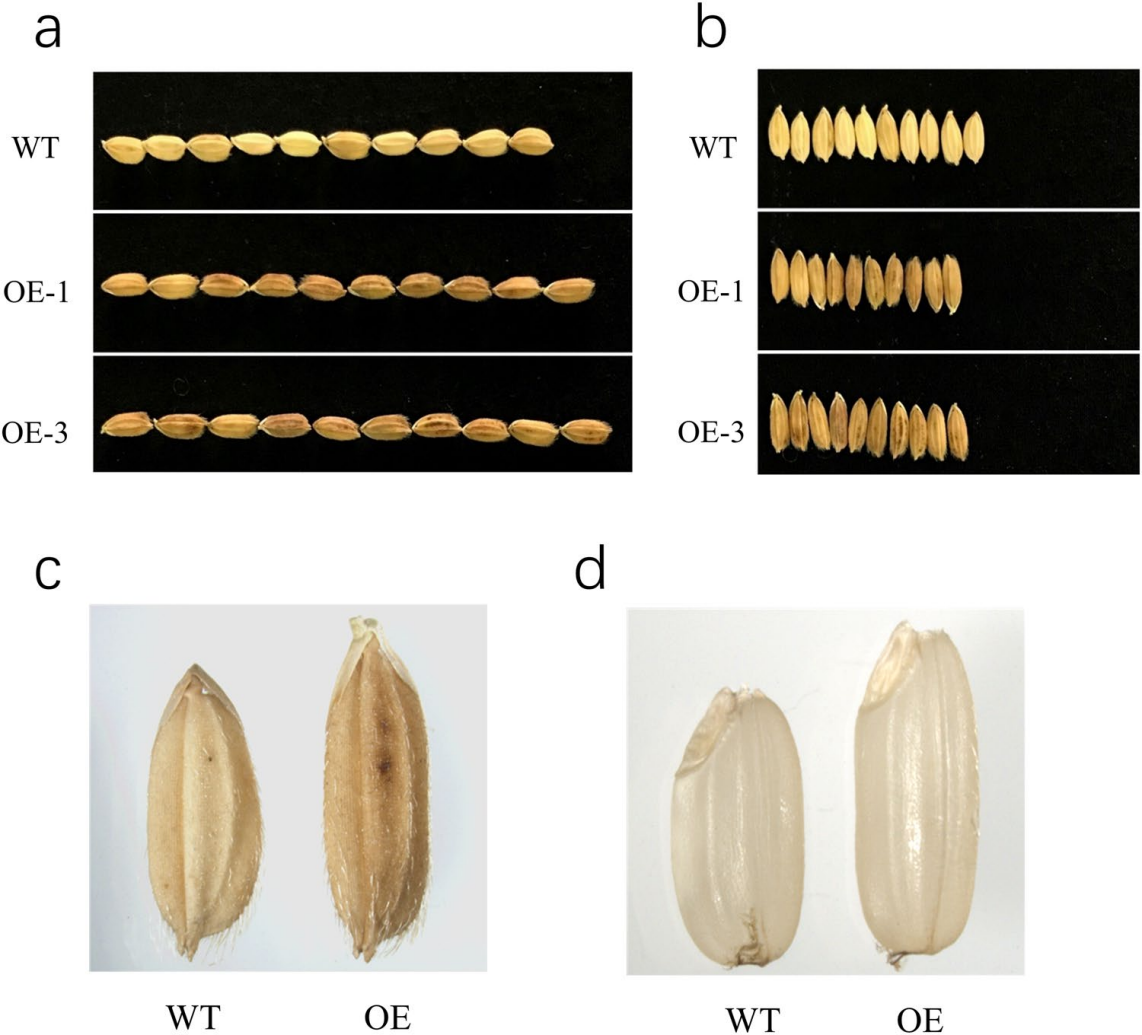
Seed phenotypes of WT and *OsSCL30-OE* of rice. (**a**) Grain length. (**b**) Grain width. (**c**) Glume color. (**d**) Endosperm color.

Compared with WT, *OsSCL30-OE* had obvious difference in grain size. Among them, grain length was significantly longer than WT, increasing by 13.5%, grain width was significantly smaller than WT, decreasing by 13.2%, grain thickness was significantly smaller than WT, decreasing by 4.3%, and 1000-grain weight was slightly larger than WT, but did not reach a significant level. In addition, the color of the glume of *OsSCL30-OE* was darker than that of WT, and the color of the endosperm was cloudier (Fig. 8). The results of field trait measurement showed that compared with WT, the plant height, tiller number, effective spikes and yield per plant of *OsSCL30-OE* were significantly reduced by 8.3%, 45.5%, 46.7% and 49.9%, respectively (Table 1).

**Table 1:** Agronomic traits of WT and *OsSCL30-OE* of rice.

Traits	WT	OE	Growth rate (%)
Grain length (mm)	0.74 ± 0.001	0.84 ± 0.012	13.5**
Grain width (mm)	0.38 ± 0.017	0.33 ± 0.013	− 13.2**
Grain thickness (mm)	0.23 ± 0.005	0.22 ± 0.008	− 4.3**
Thousand-kernel weight (g)	26.6 ± 0.351	26.8 ± 0.400	0.75
Tiller	22 ± 0.834	12 ± 0.463	− 45.5**
Plant height (cm)	96 ± 1.188	88 ± 1.165	− 8.3**
Yield per plant (g)	40.5 ± 0.865	20.3 ± 0.968	− 49.9**
Effective spikes	15 ± 0.622	8 ± 0.678	− 46.7**

The data in the table are mean ± 10 standard deviations; Asterisks indicate significant differences between WT and *OsSCL30-OE* (*P < 0.05, **P < 0.01).

## Discussion

Abiotic stresses such as drought, salt and extreme temperatures have a huge impact on world agricultural production. Plants typically respond and adapt to these stresses at the levels ranging from molecular to cellular and organ levels, encompassing a range of physiological and biochemical processes^35–38^. Understanding the complexity of the mechanisms by which plants respond to abiotic stress is crucial for developing high-yielding crops. The results of this study showed that *OsSCL30* overexpressing plants had reduced tolerance to low temperature, drought and salt.

The SR proteins are nucleophosmin proteins with characteristic Ser/Arg-rich domains and one or two RNA recognition motifs^39^.They are a very important class of splicing regulators and can be divided into six subfamilies according to their structural characteristics, namely: SR, RSZ, SC, SCL, RS2Z and RS subfamilies, of which SCL, RS2Z and RS are three subfamilies unique to plants^34,40^. The SR protein family in plants had been confirmed to have a role of alternative splicing in stress responses^41^. The expression of *AtSR45a* was induced by strong light exposure, and the expression of *AtSR30* was increased by strong light exposure and plastoquinone (PQ) or salinity treatment, and decreased by low temperature^42^. Overexpression of *Populus trichocarpa PtSCL30* in *Arabidopsis* reduced cold tolerance, possibly due to alternative splicing (AS) changes in genes critical for cold tolerance such as *ICE2* and *COR15A*^43^. The cassava *MeSCL30a* overexpression lines were hypersensitive to salt and drought stress, and had lower germination and greening rates^27^. In the present study, the *OsSCL30* (Os12g38430) (SCL subfamily) codes for the protein located in the nucleus and contains an N-terminal RNA recognition sequence. Low temperature, drought and salt stresses all decreased the expression of *OsSCL30*, with drought stress having the greatest effect on the expression of *OsSCL30* (Fig. 2a–f), indicating that *OsSCL30* may be involved in the abiotic stress responses. The overexpression of *OsSCL30* reduced the survival rate of transgenic plants under low temperature, drought and salt stresses (Figs. 3, 4 and 5). Under stress, the expression of positive regulatory genes in stress-related response in *OsSCL30-OE* was down regulated compared with WT (Figs. 3, 4 and 5). The overexpression of *OsSCL30* affected the expression of SR genes (*OsSCL30A*, *OsSCL25*, *OsSCL26*, *OsSCL28* and *OsSCL57*) of other SCL subfamilies in the transgenic line, and the expression of *OsSCL30A* increased most significantly (Fig. 7m). These results suggest that *OsSCL30* may participate in the splicing of mRNAs, produce proteins with changed function and structure, and negatively regulate the tolerance of plants to low temperature, drought and saline alkali. In addition, *OsSCL30* may have crosstalk with other members of the family and produce cumulative effects, thus affect the tolerance of transgenic plants to abiotic stress.

Plants subjected to abiotic stress accumulate ROS^44^, that can cause oxidative damage to cell membranes, proteins, DNA molecules, etc^45–47^. The activities of antioxidant enzymes SOD, CAT and POD, as well as membrane lipid peroxidation (generating MDA) can reflect the degree of damage to plants caused by stress to a certain extent. SOD can scavenge superoxide anion free radicals, CAT and POD can catalyze the decomposition of H_2_O_2_ into H_2_O and O_2_, which can help plants resist peroxidation and is positively related to the tolerance of various stresses^48–50^. As a product of ROS lipid peroxidation, MDA is often used to indicate a degree of cell membrane lipid peroxidation^51,52^. Overexpression of *Cassava MeSR34* enhanced salt stress tolerance in transgenic Arabidopsis by maintaining ROS homeostasis and affecting the CBL-CIPK pathway^53^; Overexpression of *PtSC27* in *Populus trichocarpa* enhanced salt tolerance by regulating ROS accumulation in transgenic Arabidopsis and reduces the sensitivity of transgenic plants to exogenous ABA^54^. In the study presented here, the NBT staining results of *OsSCL30-OE* and WT before the three stress treatments were similar, and the NBT-stained leaves of *OsSCL30-OE* after stress treatment showed more black spots than WT (Fig. 6a), indicating that the *OsSCL30*-overexpressing lines accumulated more ROS. Furthermore, the activities of antioxidative enzymes under non-stress conditions were similar in the *OsSCL30*-overexpressing lines and WT, but under three stress conditions (low temperature, drought, high salt), the activities of SOD, POD and CAT were lower in the *OsSCL30* overexpression lines than WT (Fig. 6c–e). The content of MDA was higher in the *OsSCL30*-overexpressing lines than WT (Fig. 6b). Under normal conditions, there was no significant difference in the expression of *OsRbohA*, *OsCu-ZnSOD2*, *OsPOD* and *OsCATA* in WT and *OsSCL30-OE*. However, under stress treatment, compared with WT, the expression of *OsRbohA* in *OsSCL30-OE* increased in varying degrees (Fig. 7a–c), and the expression of *OsCu-ZnSOD2*, *OsPOD* and *OsCATA* decreased in varying degrees (Fig. 7d–l). These findings suggested that overexpression of *OsSCL30* may alter the splicing pattern of pre-mRNA to generate proteins with multiple functions and structures, so as to regulate ROS scavenging activity, cause transgenic rice plants to suffer more severe membrane damage and reduce their tolerance to cold, drought and salt stress.

The *OsSCL30* was localized mainly in the nucleus, with tissue-specific expression (higher in leaves and leaf sheaths than roots, stems and spikelets). Under stress, *OsSCL30-*overexpression resulted in more severe membrane damage in transgenic rice plants by decreasing the reactive oxygen species scavenging activity, thereby reduced tolerance to cold, drought and salt stress. The results of field trait measurement showed that the effective spikes and yield per plant of *OsSCL30-OE* were significantly lower than those of WT, and the reduction ratio of yield per plant was the most obvious. Characterization of the negative role of *OsSCL30* in rice stress provides a theoretical basis for breeding more stress-tolerant rice using CRISPR/Cas9 gene modification system in the future.

## Materials and methods

### Plant materials and growth conditions

In this study, the japonica rice Nipponbare *(Oryza sativa* ssp. *japonica* 'Nipponbare') was used as the experimental material. We have been granted permission to collect *Oryza sativa* ssp. *japonica* 'Nipponbare'. Transgenic rice plants were obtained by agrobacterium tumefaciens mediated genetic transformation^55^. The seeds were soaked in water for 1 day, then surface sterilized by soaking in 2% v/v NaClO for 30 min and rinsing with sterile water. Afterwards, seeds were spread on moist filter paper in a Petri dish to promote germination. When the shoots grew to 5 mm, the seedlings were transplanted into rice nutrient solution and grown at 28 °C/22 °C and 16 h light/8 h dark cycle^56^. To study the effect of abiotic stress conditions on the expression of *OsSCL30*, after 2 weeks of culture, uniformly sized seedlings were selected for stress treatments, including cold (4 °C), salt (150 mM NaCl) and drought (15% w/v PEG 6000). All the experiments were performed in accordance with relevant guidelines and regulations.

### Total RNA extraction and cDNA synthesis

Fresh leaves were sampled from the normal and stress treatments, snap-frozen in liquid nitrogen, and used for total RNA extraction according to the operating instructions of the RNA extraction kit (BioFlux). RNA was reverse transcribed into cDNA using a reverse transcription kit (TaKaRa) and was stored at –20 °C.

### Quantitative analysis of *OsSCL30* under abiotic stress

Two-week-old rice seedlings were subjected to cold, drought and salt stress treatments, and leaf tissue was harvested at the indicated times for extraction of total RNA and reverse transcription. This cDNA was diluted and used as a template to analyze the gene expression of *OsSCL30* under abiotic stress. Primers were designed for *OsSCL30* gene and the rice reference gene UBQ (ubiqutin) using Primer Premier 5 software (UBQ-F:5′-AACCAGCTGAGGCCCAAGA-3′; UBQ-R:5′-ACGATTGATTTAACCAGTCCATG-3′; *OsSCL30*-qRT -F:5′-GTCTCGTTCCCGTTCTC-3′; *OsSCL30*-qRT -R:5′-GTAGTCATCTCGCCGTCT-3′).

### Analysis and cloning of the *OsSCL30* gene

To obtain the *OsSCL30*-overexpression vector, the CDS sequence of *OsSCL30* was obtained from the Rice Genome Database (rapdb). The primer for *OsSCL30* gene was designed using software Primer Premier 5 (*OsSCL30*-F:5′- tggagaggacagcccaagcttATGAGGAGGTACAGCCCACCA -3′; *OsSCL30*-R:5′- gtaccgaattcccggggatccTCAGTCGCTGCGGGCAGG-3′). The amplified target fragments were purified and recovered with a gel recovery kit. The expression vector (D-163 + 1300) was double digested with *Hind* III and *BamH* I endonucleases, followed by recombination to construct an *OsSCL30* overexpression vector.

### Analysis and cloning of promoter

The sequence information about 1.5 kb upstream of the *OsSCL30* open reading frame was obtained in NCBI (https://www.ncbi.nlm.nih.gov/), and then passed through PlantCare (http://bioinformatics.psb.ugent.be/webtools/plantcare/html/) for analysis of cis-acting elements in the *OsSCL30* promoter.

### Genetic relationship analysis of *OsSCL30* gene

In order to understand the phylogenetic relationships of *OsSCL30* sequences, species with high amino acid sequence similarity were selected and clustered by software MEGA5.0.

### Subcellular localization of *OsSCL30*

Seeds of the *OsSCL30-GFP* transgenic plants were germinated in the dark, stained with nuclear dye (DAPI), and observed using confocal microscopy.

### Transgenic plants treated with abiotic stress

To study the effect of low temperature on overexpressing plants, we cultured 2-week-old plants at 4 °C for 7 days and then allowed them to recover at 28 °C for 12 days. To study the effect of drought on overexpressing plants, we cultured 2-week-old plants at 18% w/v PEG 6000 for 8 days followed by recovery at 28 °C for 12 days. To study the effect of high salt on overexpressing plants, we cultured 2-week-old plants in 150 mM NaCl for 6 days and then allowed them to recovery at 28 °C for 12 days. For all stress treatments plants were grown in the rice nutrient solution.

### NBT staining

Two-week-old seedlings were treated at 4 °C, 20% w/v PEG 6000, or 150 mM NaCl for 2 days, respectively, and then stained. Nitro blue tetrazolium (NBT) staining was performed using the published protocols^57^.

### Measurement of the physiological parameters

Physiological parameters were determined after 2-week-old seedlings were treated for 2 days at 4 °C, 20% w/v PEG 6000 or 150 mM NaCl. The MDA content was determined by spectrophotometry^58^. The activities of antioxidant enzymes superoxide dismutase (SOD), peroxidase (POD) and catalase (CAT) were determined as described elsewhere^59^.

### Water loss rate

The measurement of water loss rate was as described by predecessors^60^. The leaves of 14-day-old rice seedlings were sampled at room temperature. The leaves were placed on filter paper on the experimental bench and weighed at specified time. Then calculate the percentage of water loss. Three biological repeats were performed on each line.

### Statistical analysis

All experiments were repeated three times, and the results were consistent. The data were processed and analyzed by the t-test, and the difference was statistically significant at *P* < 0.05(*) or *P* < 0.01(**).

## Supplementary Information

Supplementary Information.

## Data Availability

All data generated or analysed during this study are included in this published article.
