# Supplementary material for: OsSCL30 overexpression reduces the tolerance of rice seedlings to low temperature, drought and salt
Source: Sci Rep. 2022 May 19;12:8385. doi: 10.1038/s41598-022-12438-4 (PMC9120446; doi:10.1038/s41598-022-12438-4)
Supplement: Supplementary file 1 — Supplementary Information. [file 41598_2022_12438_MOESM1_ESM.pdf]

OsSCL30 overexpression reduces the tolerance of rice seedlings to low temperature, drought and salt

Jia Zhang [a1], Yihao Sun [a1], Zhanmei Zhou [a], Yifan Zhang [a], Yanmei Yang [a], Xiaofei Zan [a], Xiaohong Li [a], Jiale Wan [a], Xiaoling Gao [a], Rongjun Chen [a], Zhengjian Huang [a], Lihua Li [a] *, Zhengjun Xu [a] *

[a] Crop Ecophysiology and Cultivation Key Laboratory of Sichuan Province, Rice Research Institute of Sichuan Agricultural University, Chengdu, Sichuan 611130, China.

[1]These authors contributed equally to this work.

* Correspondence:mywildrice@aliyun.com（Z.J.X）;lilihua1976@tom.com（L.H.L）

Table S1. Primers used in this study.

**Primers for generating DNA vectors (The underline showed the restriction enzyme sites)**			
Gene name (ID)	Primer Sequence (5'-3') (Forward Primer/Reverse primer)	Bone vector	Experimental purpose
OsSCL30 (LOC_Os12g38430)	tggagaggacagcccaagcttATGAGGAGGTACAGCCCACCA / gtaccgaattcccggggatccTCAGTCGCTGCGGGCAGG	D-163+1300	*OsSCL30* overexpression vector
	tggagaggacagcccaagcttATGAGGAGGTACAGCCCACCA /ctcaccatgaccggtggatccTCGCTGCGGGCAGGGGAA	D-163+1300: pAcGFP1-N1	*OsSCL30*-GFP fusion protein

**Primers for qRT-PCR**		
Primers Name	Primer Sequence (5'-3') (Forward Primer/Reverse primer)	Gene ID
UBQ(ubiqutin)-F/R	AACCAGCTGAGGCCCAAGA / ACGATTGATTTAACCAGTCCATG	LOC_Os03g13170
OsSCL30-qRT -F/R	GTCTCGTTCCCGTTCTC / GTAGTCATCTCGCCGTCT	LOC_Os12g38430
OsPOD - qRT -F/R	AACGCAACCACCAAGCCG / CCTCGATCATGCCCATCTTGA	LOC_Os01g73200
OsCATA- qRT -F/R	CCCCAAGGTCTCCCCTGA / AACGACTCATCACACTGGGAGAG	LOC_Os02g02400
OsCu-ZnSOD2- qRT -	TGACACCACTAATGGCTGC/ CTAACCCTGGAGTCCGATGA	LOC_Os07g46990

F/R		
OsRbohA- qRT -F/R	GAGCGCGTCTGCCAATAAAC / TCAATGTAGCCGAGCCCTTC	LOC_Os01g53294
OsCBF2- qRT -F/R	TACGGCAACATGGACTTCGA/ GCCCATCCCGTCGTAGTAGTAG	LOC_Os06g03670
OsCBF3- qRT -F/R	AGCGACCTGGCGTTCG/ TCGCGTAGTACAGGTCCCA	LOC_Os09g35030
OsDREB2A- qRT -F/R	GGCTGAGATCCGTGAACCAA / GGACCATACATTGCCCTTGC	LOC_Os01g07120
OsNAC6- qRT -F/R	AGAAGGCCCTCGTCTTCTA / ACCTGAGGCTGTTCTTCTTG	LOC_Os01g66120
OsSCL30A- qRT -F/R	TCCGCAACATCCCTTTAAGC/ AACTCCACGAATGCAAACCC	LOC_Os02g15310
OsSCL25- qRT -F/R	TCGGCCATTCGGACAATTTG/ AATCCAAATCCACGCGGTTC	LOC_Os07g43950
OsSCL26- qRT -F/R	AGCACTATTCAAGCAGGTCTCC/ TCACCGGCTAACAGAAAGAGAC	LOC_Os03g25770
OsSCL28- qRT -F/R	GAGCAATTTGGCCCTGTAAAGG/ AATTCTTGCTTGGCCACTGC	LOC_Os03g24890
OsSCL57- qRT -F/R	AGCGCGATATCACATGAACC/ TGTTCGGCACTCTTCCTTTC	LOC_Os11g47830
